# Characterization of the complete plastid genome of *Psammosilene tunicoides* (Caryophyllaceae), an endangered medical herb endemic to south-western China

**DOI:** 10.1080/23802359.2019.1659120

**Published:** 2019-09-02

**Authors:** Yuling Li, Jiuxiang Huang, Gang Yao

**Affiliations:** Guangdong Key Laboratory for Innovative Development and Utilization of Forest Plant Germplasm, College of Forestry and Landscape Architecture, South China Agricultural University, Guangzhou, China

**Keywords:** Caryophyllaceae, phylogenomics, plastid genome, *Psammosilene*

## Abstract

*Psammosilene tunicoides* is an endangered medical herb endemic to south-western China. In this study, the complete plastid genome of the species was characterized and assembled using the next-generation DNA sequencing method. The plastid genome is 153,978 bp in length, including a large single copy (LSC) region of 83,981 bp and a small single copy (SSC) region of 17,489 bp, which were separated by a pair of inverted repeat (IR) regions of 26,254 bp. The genome encoded 112 unique genes, including 78 protein-coding genes, four ribosomal RNA genes, and 30 transfer RNA genes. The overall GC content of the whole genome is 36.49%. The phylogenetic analysis based on 17 plastid genome of Caryophyllaceae revealed that *P. tunicoides* nested within the tribe Caryophylleae with strong support value.

*Psammosilene* W.C. Wu & C.Y. Wu is a monotypic genus endemic to south-western China and belongs to the tribe Caryophylleae, family Caryophyllaceae (Lu et al. 2001; Greenberg and Donoghue 2011). The species *P. tunicoides* W.C. Wu & C.Y. Wu is a perennial herb and grows usually in rocky mountain slopes, dry pastures, and calcareous rock crevices, at the elevation of 900‒3800 m (Tang 1996; Lu et al. 2001). It is also an important plant used in traditional Chinese medicine, and its dried roots usually have been used to treat haemostasis, rheumatism and trauma haemorrhage (Zhang et al. 2011; Li et al. 2016). Due to the rapid decline of the wild populations, *P. tunicoides* is classified as a rare and endangered species in the Chinese Plant Red Book (Fu and Jin 1992).

Total genomic DNA of *P. tunicoides* was extracted from mature leaves of an individual collected from Yunnan province (China; N25°18′, E102°75′) with the modified CTAB method (Doyle and Doyle 1987). Voucher specimen (*G. Yao YGYN2015071101*) was deposited in the Herbarium of South China Botanical Garden, Chinese Academy of Sciences (IBSC). The DNA extracted was sequenced using the Illumina HisSeq 2500 Sequencing System. Reads were assembled using the software GetOrganelle (Jin et al. 2018) and all of the genes in the plastid genome were annotated with PGA (Qu et al. 2019). The accession number MN136196 was obtained from GenBank for the annotated plastid genome of *P. tunicoides*.

Structural analysis of the complete plastid genome of *P. tunicoides* exhibits a typical quadripartite circular structure with 153,978 bp in length. The genome is composed by a large single-copy (LSC) region of 83,981 bp, a small single-copy (SSC) region of 17,489 bp, and a pair of inverted repeat (IRa and IRb) regions of 26,254 bp. The genome encoded 112 unique genes, including 78 protein-coding genes, four ribosomal RNA genes (rrn4.5, rrn5, rrn16, and rrn23) and 30 transfer RNA genes, whereas the pseudogenization of *infA* was detected in plastid genome of *P. tunicoides*. Seventeen genes, including six protein-coding genes (*ndhB*, *rpl2*, *rpl23*, *rps12*, *rps7*, and *ycf2*), four ribosomal RNA genes, and seven transfer RNA genes (trnA-UGC, trnI-CAU, trnI-GAU, trnL-CAA, trnN-GUU, trnR-ACG, trnV-GAC) were found to be duplicated in IR regions. Fourteen genes contained one intron and two genes contained two introns. The overall GC content of *P. tunicoides* plastid genomes is 36.49% (LSC, 34.24%; SSC, 30.05%; IRs, 42.25%).

In this study, *P. tunicoides* and sixteen published complete plastid genome of Caryophyllaceae were used to construct a phylogenetic tree. Seven species from Achatocarpaceae, Aizoaceae and Amaranthaceae s.l. were selected as outgroups, based on phylogenetic relationships constructed recently (Yao et al. 2019). Phylogenetic result showed that all of the Caryophyllaceae members formed a well-supported clade and *P. tunicoides* clustered within the tribe Caryophylleae with 100% bootstrap support value (Figure 1), in agreement with previously published phylogenetic study (Greenberg and Donoghue 2011). The complete plastid genome sequence of *P. tunicoides* will provide a useful resource for the conservation genetics of the species.

**Figure 1.**
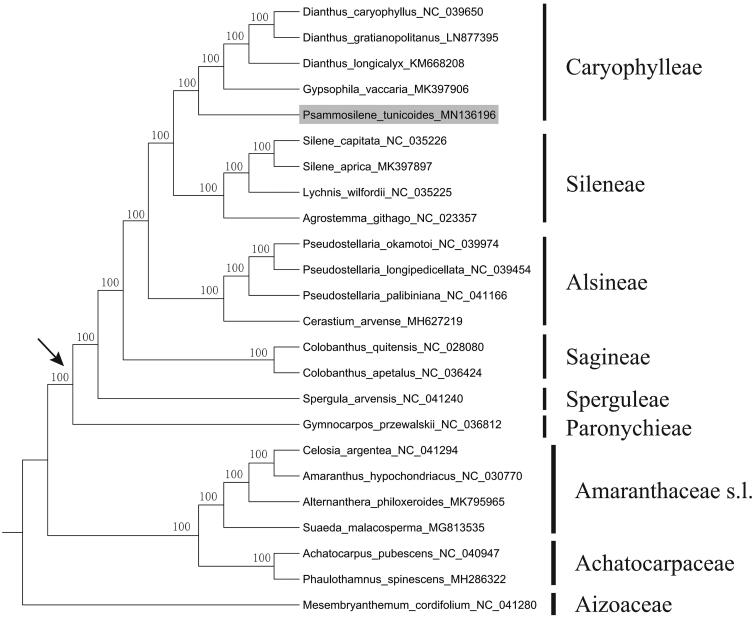
Maximum-likelihood tree of Caryophyllaceae inferred from 78 protein-coding genes and four ribosomal RNA genes of 24 plastomes (including outgroups from Achatocarpaceae, Aizoaceae and Amaranthaceae s.l.). Bootstrap values are indicated above branches. The crown node of Caryophyllaceae is shown by an arrowhead. The species *Psammosilene tunicoides* is marked with gray background.
